# The keystone gut species *Christensenella minuta* boosts gut microbial biomass and voluntary physical activity in mice

**DOI:** 10.1128/mbio.02836-23

**Published:** 2023-12-22

**Authors:** Tanja Akbuğa-Schön, Taichi A. Suzuki, Dennis Jakob, Dai Long Vu, Jillian L. Waters, Ruth E. Ley

**Affiliations:** 1 Department of Microbiome Science, Max Planck Institute for Biology Tübingen, Tübingen, Germany; 2 Mass Spectrometry Facility, Max Planck Institute for Biology Tübingen, Tübingen, Germany; 3 Cluster of Excellence EXC 2124 Controlling Microbes to Fight Infections, University of Tübingen, Tübingen, Germany; University of Hawaii at Manoa, Honolulu, Hawaii, USA

**Keywords:** microbiome, *Christensenella*, energy

## Abstract

The gut bacteria of the family *Christensenellaceae* are consistently associated with metabolic health, but their role in promoting host health is not fully understood. Here, we explored the effect of *Christensenella minuta* amendment on voluntary physical activity and the gut microbiome. We inoculated male and female germ-free mice with an obese human donor microbiota together with live or heat-killed *C. minuta* for 28 days and measured physical activity in respirometry cages. Compared to heat-killed, the live-*C. minuta* treatment resulted in reduced feed efficiency and higher levels of physical activity, with significantly greater distance traveled for males and higher levels of small movements and resting metabolic rate in females. Sex-specific effects of *C. minuta* treatment may be in part attributable to different housing conditions for males and females. Amendment with live *C. minuta* boosted gut microbial biomass in both sexes, immobilizing dietary carbon in the microbiome, and mice with high levels of *C. minuta* lose more energy in stool. Live *C. minuta* also reduced within and between-host gut microbial diversity. Overall, our results showed that *C. minuta* acts as a keystone species: despite low relative abundance, it has a large impact on its ecosystem, from the microbiome to host energy homeostasis.

**IMPORTANCE:**

The composition of the human gut microbiome is associated with human health. Within the human gut microbiome, the relative abundance of the bacterial family *Christensenellaceae* has been shown to correlate with metabolic health and a lean body type. The mechanisms underpinning this effect remain unclear. Here, we show that live *C. minuta* influences host physical activity and metabolic energy expenditure, accompanied by changes in murine metabolism and the gut microbial community in a sex-dependent manner in comparison to heat-killed *C. minuta*. Importantly, live *C. minuta* boosts the biomass of the microbiome in the gut, and a higher level of *C. minuta* is associated with greater loss of energy in stool. These observations indicate that modulation of activity levels and changes to the microbiome are ways in which the *Christensenellaceae* can influence host energy homeostasis and health.

## INTRODUCTION

The gut microbiome is composed primarily of bacterial cells representing a diversity of phyla. Of these, the Firmicutes is the most prevalent across individuals and also often the most abundant within individual microbiomes (1, 2). The Firmicutes phylum itself includes a diversity of taxa, including the bacterial family *Christensenellaceae*, a highly prevalent group within the human gut microbiome (3). The *Christensenellaceae* family of bacteria is intriguing for several reasons. First, individuals have a genetic predisposition to the proportion of these bacteria in the gut (e.g., they are heritable). Indeed, the genotype of the host is estimated to influence 30%–60% of the variation between individuals in the relative abundance of *Christensenellaceae* (3–6). Second, this family is central to co-occurrence networks with other taxa (3) and co-occurs with methanogenic archaea in particular (7–9). The co-occurrence of *Christensenellaceae* with methanogens likely results from cross-feeding through interspecies hydrogen transfer, as different species of *Christensenella* can support the growth of methanogens through production of hydrogen gas (7). Given their metabolic role in the community and their hub status in co-occurrence networks, the *Christensenellaceae* can be considered a keystone taxon (10). Third, the relative abundance of *Christensenellaceae* in the gut microbiome is consistently associated with metabolic health (7, 10). How the abundance of *Christensenellaceae* in the gut is connected to host health remains to be fully elucidated.

Goodrich et al. (3) first observed that *Christensenellaceae* was significantly enriched in lean versus obese individuals (3). Since this first observation, multiple studies have connected the abundance of *Christensenellaceae* to metabolic health, and in particular to a healthy body mass index (summarized in Fig. S1), including in studies published before the family was named (11). Studies in mice have suggested a causal relationship between the *Christensenellaceae* and host health. In a first study using fecal transplants to germ-free (GF) mice, we showed that addition of live *Christensenella minuta* to a gut microbiome obtained from an obese human donor reduced adiposity gains of recipient mice compared to heat-killed or vehicle control (3). Corroborating these observations, administration of *C. minuta* or *Luoshenia tenuis* to mice fed a high-fat diet was also shown to reduce adiposity gains compared to vehicle control (12, 13). Moreover, administration of live *C. minuta* to mice on a high-fat diet decreased feed efficiency (body weight gain per total ingested calories) compared to pasteurized and vehicle control (13). Reduced feed efficiency can arise from a lower energy harvest from food or a higher host energy expenditure, both of which have been shown to be under the influence of the gut microbiome (14–22). Additionally, several studies have associated physical activity with *Christensenellaceae* in humans (23–25) and in animal studies (17, 26). Although these associations between the *Christensenellaceae* and physical activity suggest a causative link, this has not been tested directly.

Here, we tested whether administration of *C. minuta* can influence voluntary physical activity in a mouse model. We addressed this question with a well-powered fecal transplant experiment based on results from our previous study design (3). We inoculated 184 male and female GF mice with human feces from an obese donor and amended the human feces with live or heat-killed *C. minuta*. We assessed individual aspects of energy homeostasis, including food intake, activity, behavior, and energy expenditure, using a behavioral phenotyping respirometry cage system. Our results show that compared to heat-killed control, treatment with live *C. minuta* resulted in lower host feed efficiency, higher host activity, and higher energy expenditure. Furthermore, amendment with live *C. minuta* led to higher gut microbial biomass, loss of energy in stool, lower microbial α-diversity, and alterations in host and microbial metabolism.

## RESULTS

### Mice with live *C. minuta* show lower feed efficiency

**Fig 1:**
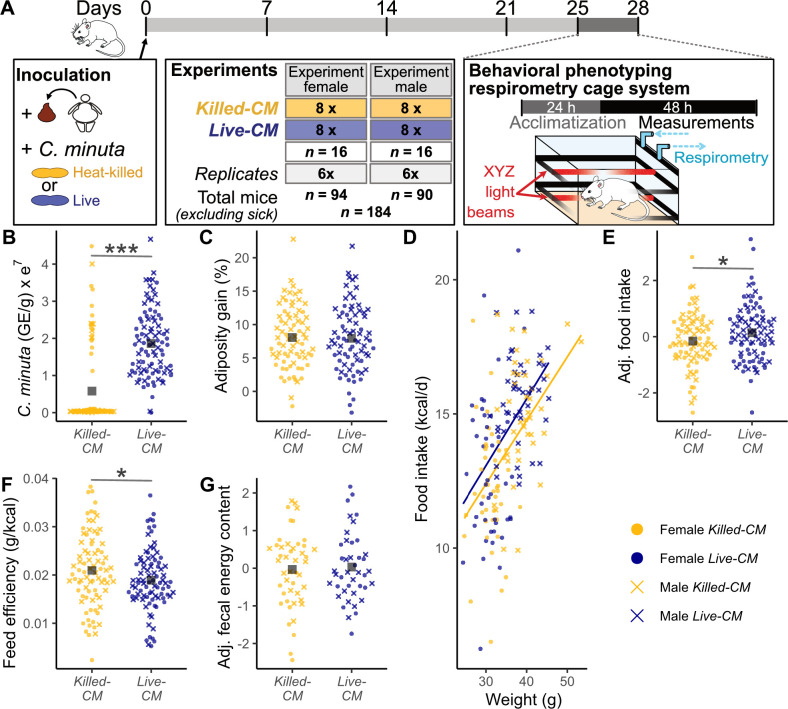
Effects of live or heat-killed *C. minuta* amendment to fecal transplants to GF mice on *C. minuta*, murine adiposity, feed efficiency, and fecal energy loss 4 weeks post-inoculation. (**A**) Overview of the experimental set-up. Inocula consisted of a slurry derived from a fecal sample from an obese human donor amended with living (Live-CM) or heat-killed *C. minuta* (Killed-CM). Each experiment included 16 mice of one sex, with 8 mice per treatment group; each experiment was replicated six times. During the first 25 days, mice were housed in groups of four (females) or singly (males) at 22°C. After 25 days, mice were transferred to a sterilized behavioral phenotyping respirometry cage system (Promethion Sable Line, Nevada, USA) and housed singly at a temperature of 26°C. The first 24 h were considered as acclimatization and excluded from the analysis. (**B**) Quantification by qPCR of *C. minuta* in murine cecal contents, normalized by wet weight of cecal contents used for DNA extraction collected on day 28 post-inoculation. (**C**) Percent change in adiposity from day 0 to day 25 post-inoculation. (**D and E**) Average daily food intake (FI) from day 25 to day 28 post-inoculation: (**D**) FI raw values correlated with body weight and (**E**) FI residuals adjusted for sex, batch, and weight. (**F**) Feed efficiency (FE) over the duration of the experiment calculated using weight gain from day 0 to day 28 post-inoculation and daily food intake. (**G**) Residuals of daily energy excreted via feces (fecal energy) measured via bomb calorimetry adjusted for sex, batch, and weight. Asterisks indicate statistical significance of the linear mixed model correcting for sex, batch, and (**D, E, and G**) mouse weight. *P < 0.1; **P < 0.01; and ***P < 0.001. Adj. = adjusted and GE = genome equivalents.

We gavaged 5–6-week-old GF Swiss Webster mice with an anoxic fecal slurry made from a single stool sample obtained from an obese human donor selected for its low levels of *C. minuta*. The slurry was amended with either live (Live-CM) or heat-killed *C. minuta* (Killed-CM) (10^10^ CFUs/dose/mouse) prior to gavage. Male mice fought and had to be single-housed for their welfare; female mice were co-housed four per cage. Mice were maintained at room temperature (22°C) and provided polysaccharide-rich chow *ad libitum*. After 25 days, we transferred mice singly to a behavioral phenotyping respirometry cage system with an ambient temperature of 26°C (Promethion Sable Line, Nevada, USA). We assessed body weight weekly and body composition at inoculation and at day 25 post-inoculation. For each experiment, we used 16 mice of one sex, 8 for each treatment group. For each sex, we replicated the experiment six times, resulting in a combined sample size of 90 male and 94 female mice (Fig. 1A).

To confirm that live *C. minuta* amendment resulted in higher levels of *C. minuta* relative to the Killed-CM control, we quantified *C. minuta* DNA content in ceca at day 28 post-inoculation by qPCR using species-specific primers (27). As anticipated, administration of live *C. minuta* resulted in higher levels of *C. minuta* DNA content compared to heat-killed controls [Live-CM = 18.9e^6^ ± 1e^6^ genome equivalents (GE); Killed-CM = 5.8e^6^ ± 2.3e^6^ GE; *P* = 2.2e^−4^, Fig. 1B].

We evaluated how administration of live *C. minuta* impacted body composition, food intake, and feed efficiency. We observed no differences between treatments for mean gains in adiposity (Fig. 1C) or weight (Fig. S2B). However, we detected a trend for 5% higher food intake in the Live-CM versus Killed-CM mice for both sexes (Live-CM = 14.3 ± 0.2 g; Killed-CM = 13.6 ± 0.2 g; *P* = 0.055, Fig. 1D and E). We estimated feed efficiency by dividing weight gain by food intake. We observed 10.5% lower feed efficiency for the Live-CM versus Killed-CM treated mice (Live-CM *=* 1.72e^−2^ ± 0.11e^−2^ g/kcal; Killed-CM *=* 1.92e^−2^ ± 0.11e^−2^ g/kcal; *P* = 0.027, Fig. 1F). To test if live *C. minuta* affected energy loss in stool, we quantified the energy content of all feces excreted between days 25 and 28 post-inoculation by bomb calorimetry. We observed no difference in daily fecal energy loss (*P* = 0.48, Fig. 1G) or fecal mass (*P* = 0.63, Fig. S2C) between treatments.

### *C. minuta* amended mice displayed higher physical activity and metabolic energy expenditure in a sex-dependent manner

We measured activity and energy expenditure using behavioral phenotyping respirometry cages, in which physical activity is assessed automatically by beam breaks and aerobic energy expenditure is measured by respirometry. We obtained two related activity metrics monitored by the light-beam-break system: physical activity (number of beam breaks in the *XYZ*-axes) and distance traveled (within the *XY*-axes in meters). Physical activity represents all movements independent of movement size, including scratching, grooming, rearing, and distance traveled. In contrast, distance traveled is estimated solely on movement in the *XY*-axes. Thus, a mouse with high levels of fidgety behaviors but that runs very little may exhibit physical activity levels similar to a mouse that runs more but fidgets less. We considered the first 24 h to be a period of acclimatization to the new cage environment (28) and excluded them from analysis (Fig. 1A).

**Fig 2:**
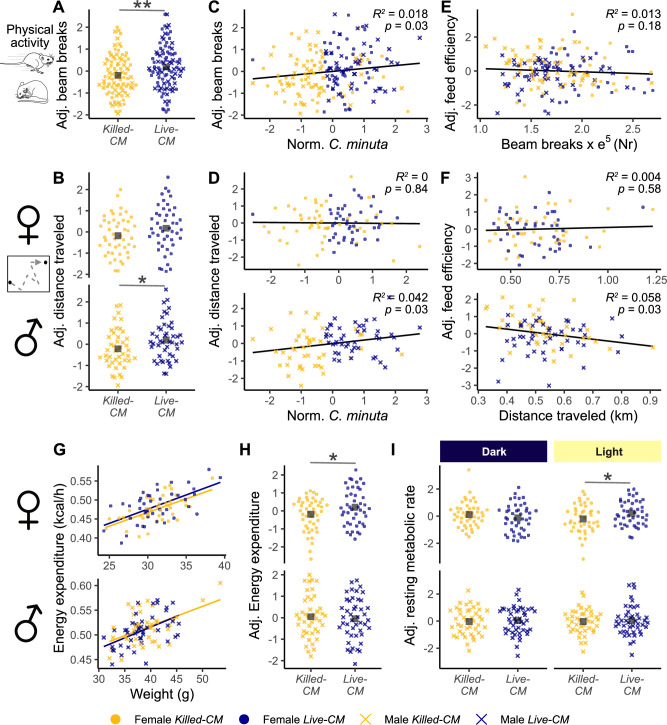
Higher physical activity and metabolic energy expenditure in mice with live *C. minuta*. (**A**) Number of beam breaks by treatment group. (**B**) Distance traveled by sex and treatment. (**C**) Beam breaks plotted against normalized and standardized *C. minuta*. (**D**) Distance traveled plotted against normalized and standardized *C. minuta*, by sex. (**E**) Feed efficiency plotted against beam breaks. (**F**) Feed efficiency plotted against distance traveled, by sex. (**G**) Average hourly energy expenditure plotted against body weight for males and females. (**H**) Energy expenditure by treatment and sex. (**I**) Resting metabolic rate during the dark and light cycles. “Adj”: data were adjusted for effects of cabinet, batch, and sex (**A–F**), and body weight, batch, and sex (**H and I**). Asterisks indicate levels of statistical significance: *P < 0.1; **P < 0.01; and ***P < 0.001. Adj. = adjusted; GE = genome equivalents; and norm. = normalized.

We observed that Live-CM amendment treatment was associated with a higher mean physical activity compared to Killed-CM in both male and female mice (6.8% higher beam breaks, Live-CM = 12e^4^ ± 5e^4^ beam breaks; Killed-CM = 11.2e^4^ ± 5e^4^ beam breaks; *P* = 0.009, Fig. 2A). In contrast, we observed sex-dimorphic treatment effects for distance traveled. Live-CM males exhibited 8.5% more distance traveled on average compared to Killed-CM males (Live-CM males = 334 ± 20 m; Killed-CM males = 308 ± 20 m; *P* = 0.02, Fig. 2B). There was no difference in mean distance traveled for females (Live-CM females = 410 ± 37 m; Killed-CM females = 415 ± 37 m; *P* = 0.75, Fig. 2B). Mean running speed was similar between treatments for both sexes (*P* = 0.2, Fig. S3A).

We also assessed the relationship between the cecal content of *C. minuta* cecal (Fig. 1B), regardless of treatment group, with physical activity metrics. We observed that physical activity was positively associated with *C. minuta* for both sexes (*R*^2^ = 0.018, *P* = 0.03, Fig. 2C). Moreover, *C. minuta* showed a trend for positive correlation with the distance traveled of male mice (*R*^2^ = 0.042, *P* = 0.03, Fig. 2D) but not of females (*R*^2^ = 4.6e^−4^, *P* = 0.84, Fig. 2D).

Physical activity was not correlated with feed efficiency (*R*^2^ = 0.006, *P* = 0.33, Fig. 2E). Males showed a negative correlation between distance traveled and feed efficiency (*R*^2^ = 0.061, *P* = 0.02, Fig. 2F), while no association was present in females (*R*^2^ = 0.004, *P* = 0.56, Fig. 2F).

We observed a higher trend for total energy expenditure in the Live-CM females compared to the Killed-CM females (Live-CM = 0.481 ± .009 kcal/h; Killed-CM = 0.47 ± .008 kcal/h; *P* = 0.06, Fig. 2G and H), but was indistinguishable between the male treatments (Live-CM = 0.51 ± .006 kcal/h; Killed-CM = 0.512 ± .006 kcal/h; *P* = 0.67, Fig. 2G and H). To evaluate if the physical activity or independent metabolic processes mediated the higher energy expenditure in females with live *C. minuta*, we examined energy expenditure assessed while the animal was physically at rest, an approximation of the resting metabolic rate (RMR). The average RMR of both sexes showed no difference between the treatment groups (females: *P* = 0.54; males: *P* = 0.58, Fig. S3B). However, when we examined each circadian cycle separately, we observed a higher RMR in the Live-CM females compared to the Killed-CM females during the light cycle (Live-CM = 0.41 ± .007 kcal/h; Killed-CM = 0.39 ± .007 kcal/h; *P* = 0.048, Fig. 2I). We observed no differences between the treatments for females during the night cycle (*P* = 0.23, Fig. 2I), nor for males during light (*P* = 0.3, Fig. 2I) or dark cycle (*P* = 0.7, Fig. 2I).

### *C. minuta* increases gut microbial biomass and remodels diversity

**Fig 3:**
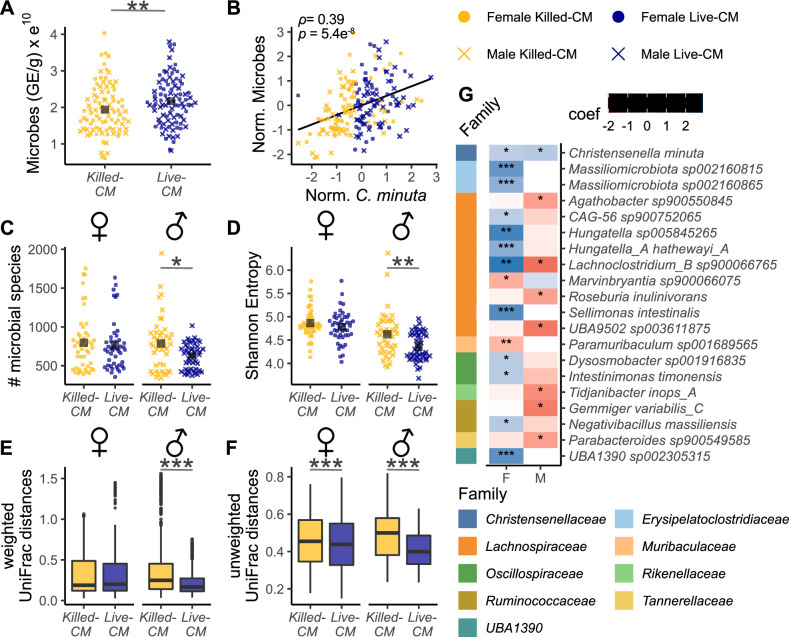
*C. minuta* effects on gut microbiome biomass and diversity. (**A**) Quantification of microbial biomass (genome equivalents) in ceca via qPCR with universal 16S rRNA primers determined by the microbial standards and normalized by wet weight of cecal contents used for the DNA extraction and plotted by treatment group. (**B**) Microbial biomass (normalized plus standardized) plotted against *C. minuta (*normalized plus standardized). (**C and D**) α-diversity of phylogenetically profiled metagenomic cecal sequences by treatments. (**E**) Intra-group weighted and (**F**) unweighted UniFrac distances of the treatments. (**G**) Differential abundance analysis of microbial species using MaAsLin2 (29) and multiple hypothesis correction with the Benjamini-Hochberg method. Coefficients indicate associations with treatments (red: negative values = higher in Killed-CM and blue: positive values = higher in Live-CM). Asterisks indicate statistical significance of (**A, C, and D**) the linear mixed model correcting for sex and batch or (**E and F**) a permuted Wilcoxon rank sum test. (**B**) *R*^2^ and *P* values of Spearman’s correlation analyses. *P < 0.1; **P < 0.01; and ***P < 0.001. Adj. = adjusted; F = females; GE = genome equivalents; Norm. = normalized; and M = males.

We performed qPCR using universal 16S rRNA primers to quantify the total microbial biomass in cecal contents. We observed that the Live-CM mice of both sexes had, on average, ~10% greater microbial biomass compared to the Killed-CM group (Live-CM = 2.2e^10^ ± 0.8e^10^ GE; Killed-CM = 1.9e^10^ ± 0.8e^10^ GE; *P* = 0.008, Fig. 3A). We detected a positive correlation between the microbial biomass in cecal contents and *C. minuta* (Spearman’s *ρ* = 0.39, *P* = 5.4e^−8^, Fig. 3B). Given the relatively low cell count for live *C. minuta*, the treatment itself did not account for the higher biomass measured in the Live-CM group compared to the Killed-CM group. Thus, the metabolic activity of *C. minuta* in the community boosted overall biomass.

Next, we assessed microbial community richness using four α-diversity metrics: number of microbial species, Faith’s phylogenetic diversity (PD), Shannon Entropy, and Pielou evenness. We observed significantly lower α-diversity in the Live-CM males compared to Killed-CM in males for the first three of these metrics (Table S1). In contrast to males, females showed no differences in α-diversity between the treatments (Fig. 3C and D; Fig. S4B and C; Table S1).

To assess how the treatments affected between-sample diversity, we applied the beta-diversity UniFrac metrics (30). Using a permuted Wilcoxon test, we observed more similar microbiomes within the Live-CM males than within the Killed-CM males, using both weighted (*P* = 2.2e^−16^, Fig. 3F) and unweighted (*P* = 2.2e^−16^, Fig. S4E) UniFrac distances. Females also had lower unweighted-UniFrac distances within the Live-CM group compared to within the Killed-CM group (*P* = 7.3e^−5^, Fig. S4E) with a similar trend for weighted-UniFrac distances (*P =* 0.6, Fig. 3F). These results indicate that live *C. minuta* amendment exerts a conforming influence on microbial diversity.

Using differential abundance analyses, we analyzed treatment effects on the absolute abundances of species. Due to the variation in biomass between mice, we normalized abundances of taxa by the total microbial genome equivalents per mouse (Fig. S4A). We observed 14 differentially abundant species between treatments in females and 8 species for males (Fig. 3G). The lists differ for males and females with the exception of two species. One is *C. minuta*, which, as expected, had a higher abundance in the Live-CM groups compared to the Killed-CM groups in both sexes (Fig. 3G). The other species observed as differentially abundant by treatment for the two sexes was *Lachnoclostridium_B*, which showed a higher relative abundance in live versus heat-killed for female mice and the reverse for males. Other taxa differed between treatments in males or females, with females showing patterns of enrichment with Live-CM and males showing enrichment of taxa in Killed-CM (Fig. 3G). Thus, even though the overall effect of Live-CM was to reduce between-microbiome variability, the specific effects on the microbiome species composition differed by sex.

### Amendment of *C. minuta* altered the host metabolome

**Fig 4:**
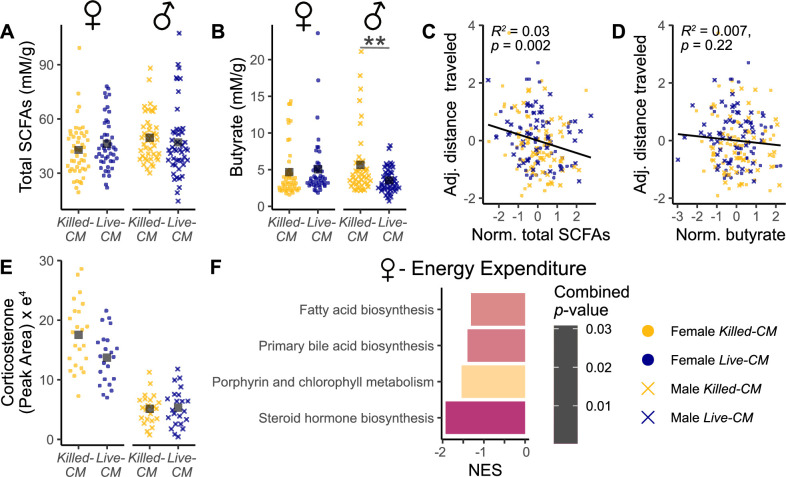
Changes of metabolism in relation to activity and energy expenditure. (**A**) For each sex, the sum of measured SCFA concentrations in murine cecal contents on day 28 post-inoculation was determined via gas chromatography-mass spectrometry. (**B**) Butyrate concentrations per sex and treatment. (**C**) Total SCFAs (normalized and standardized) plotted against adjusted distance traveled. (**D**) Butyrate (normalized and standardized) plotted against adjusted distance traveled. (**E**) Serum corticosterone levels at day 28 post-inoculation measured via liquid chromatography-mass spectrometry (LC-MS) per sex and treatment group. (**F**) Predicted metabolic pathways significantly associated with female energy expenditure including animals from both treatment groups, determined by analyzing the entire LC-MS peak spectrum of murine serum samples with XCMS (v.2.7.2) (31), MaAsLin2, including multiple hypothesis adjustment (29), and MetaboAnalyst5.0 (www.metaboanalyst.ca) (32). Pathways with a combined *P* value (GSEA and mummichog) <0.05 were considered significant. Asterisks indicate statistical significance of the linear mixed model correcting for sex and batch. (**B and D**) Marginal *R*^2^ and *P* values of linear mixed model analyses are stated in the figure. **P < 0.01. Adj. = adjusted; NES = normalized enrichment score; Norm. = normalized; and SCFAs = short-chain fatty acids.

We measured five short-chain fatty acids (SCFAs) in cecal contents (Fig. 4A and C; Fig. S5A). SCFAs trended to be higher overall in the Live-CM group compared to the Killed-CM mice in females (Live-CM: 46.3 ± 1.7 mM/g; Killed-CM: 42.8 ± 1.6 mM/g; *P* = 0.1, Fig. 4A), but showed no treatment effect in males (Live-CM = 47.2 ± 2.2 mM/g; Killed-CM = 49.4 ± 2.3 mM/g; *P* = 0.16, Fig. 4A). Analyzing SCFAs individually, butyrate was significantly lower in the Live-CM compared to Killed-CM treatment males (Live-CM = 3.5 ± 1 mM/g; Killed-CM = 5.7 ± 1 mM/g; *q* = 0.006, Fig. 4B). We observed a negative correlation between total cecal SCFAs with distance traveled (*R*^2^ = 0.032, *P* = 0.002, Fig. 4C) and with physical activity (*R*^2^ = 0.016, *P* = 0.03, Fig. S5B) in both sexes. Cecal butyrate levels did not correlate with activity measures (distance traveled: *R*^2^ = 0.007, *P* = 0.22, Fig. 4D; physical activity: *R*^2^ = 0.003, *P* = 0.43, Fig. S5D) in both sexes combined nor in each sex separately.

We next selected 11 serum compounds, known to be influenced by the gut microbiome and previously shown to affect host behavior (Table S2), for quantification by liquid chromatography-mass spectrometry (LC-MS) with matching standards (Fig. 4E; Fig. S5E). Females showed a trend for lower corticosterone, the main rodent stress hormone, in the Live-CM group compared to the Killed-CM group (Live-CM = 13.8e^4^ ± 1.1e^4^ peak area (PA); Killed-CM = energy 17.6e^4^ ± 1e^4^ PA; *q* = 0.12, Fig. 4E). We then analyzed the entire peak spectrum of the same LC-MS runs, which included peaks of the targeted metabolites as well as non-targeted, unidentified metabolites. Here we analyzed the entire LC-MS peak spectrum of serum samples of both sexes with XCMS (v.2.7.2) (31) for peak identification, MaAsLin2 (29) for differential abundance analysis adjusting for batch effects, and MetaboAnalyst5.0 (www.metaboanalyst.ca) (32) for a functional analysis. Here, the integrated mummichog algorithm predicts empirical compounds from the LC-MS peaks on the pathway level, using the idea that changes occur not in individual metabolites but in whole pathways (33). Next, using two different statistical methods, GSEA and mummichog, pathway enrichment is calculated on the predicted compounds and combined *P* values <0.05 were considered significant. With this approach of two different statistical methods, the reliability of the predicted pathway enrichment is improved (34). We observed, in females, associations between energy expenditure and pathways involved in glucocorticoid synthesis, including steroid hormone biosynthesis (*P* = 2e^−4^, Fig. 4F) and primary bile acid biosynthesis (*P* = 0.012, Fig. 4F). These associations were independent of the treatment group for female mice. The predicted compounds of each significant pathway are listed in Table S3.

### Regrouping mice by final *C. minuta* content corroborates treatment effects

In the categorical analyses above, we grouped the mice by treatment. However, our measurements of *C. minuta* cecal DNA content at 28 days post-inoculation indicated that some mice in the Killed-CM group had final levels of *C. minuta* more similar to the Live-CM treatment group, and vice versa. To assess the effect of *C. minuta* regardless of treatment group, we regrouped the mice based on *C. minuta* in the microbiome at the end of the experiments. We selected the median amount of *C. minuta* (median = 11,072,203 GE) as the threshold for partitioning of mice into the High-CM (*C. minuta* > median *C. minuta*, Fig. S6A) and Low-CM groups (*C. minuta* ≤ median *C. minuta*, Fig. S6A). These new groups were compared for energy homeostasis, as well as microbial biomass and microbiome diversity.

We observed no differences between the groups for total food intake (*P* = 0.22, Fig. S6B), but we did observe a trend for 10% lower feed efficiency in High-CM mice compared to the Low-CM group for both sexes (High-CM *=* 1.7e^−2^ ± 0.12e^−2^ g/kcal; Low-CM *=* 1.9e^−2^ ± 0.12e^−2^ g/kcal; *P* = 0.052, Fig. S6C). Furthermore, we observed 5% lower fecal energy content for High-CM mice compared to the Low-CM group for both sexes (High-CM: 3.53 ± 0.09 kcal/day; Low-CM: 3.72 ± 0.1 kcal/day; *P* = 0.066, Fig. S6D). High-CM mice tended to have higher physical activity compared to the Low-CM group in both sexes (High-CM: 12.3e^4^ ± 0.6e^4^ beam breaks; Low-CM: 11.5e^4^ ± 0.6e^4^ beam breaks; *P* = 0.06, Fig. S6E). However, the distance traveled was similar between the groups (females: *P* = 0.74, Fig. S6F; males: *P* = 0.55, Fig. S6F). We observed no difference between the groups in terms of energy expenditure measured via indirect calorimetry (data not shown).

The analysis of the microbiome data with the new groups strengthened the association between microbial biomass and *C. minuta.* High-CM mice had ~17% higher biomass compared to Low-CM mice (High-CM = 2.22e^10^ ± 0.87e^10^ GE; Low-CM = 1.89e^10^ ± 0.87e^10^ GE; *P* = 0.001, Fig. S7A). Microbial biomass and *C. minuta* did not correlate with fecal energy content (Fig. S7B and C). The High-CM mice of both sexes had reduced richness (number of microbial species *P* = 1.6e^−4^, Fig. S7D; Faith’s PD *P* = 1.3e^−4^, Fig. S7E through G; Table S4). The High-CM had more similar microbiome diversity between samples compared to the Low-CM group (weighted UniFrac *P =* 2.2e^−16^; unweighted UniFrac *P =* 2.2e^−16^, Fig. S7H and I).

## DISCUSSION

Physical exercise has been shown to influence gut microbiome composition in humans (35–37)*. C. minuta* specifically has been associated with physical activity in humans (23–25) and in rodents (17, 26). Our study tested the causality of this relationship with a well-powered fecal transplant study using both male and female mice. We observed significantly higher voluntary physical activity in mice receiving live compared to heat-killed *C. minuta*. The type of physical activity differed between male and female mice, which may be partly attributable to the different housing conditions required for male and female mice. For both sexes, live *C. minuta* drove a significant increase in gut microbial biomass and modified microbiome diversity and metabolism. Our results indicate that addition of living cells of this single species, *C. minuta*, suffices to remodel the gut microbiome and its metabolic outputs. How these changes may be linked to the altered host behavior remains to be clarified.

Our results show that live *C. minuta* amendment to a microbiome increases voluntary physical activity in male and female mice but in different ways. Administration of live *C. minuta* drove males to travel greater distances, whereas in females, live *C. minuta* induced overall higher levels of fine movement and increased their resting metabolic rate. We should note that for animal welfare reasons, the male mice were housed singly, whereas the females were housed in groups; thus, housing was confounded with sex and may play a role in the sex differences we observed for the type of movement affected by the treatments. Moreover, male and female mice differ in activity phenotypes in general (38, 39). The reasons underlying the sex differences in treatment effect on the types of voluntary activity associated with the treatment remain to be elucidated. Regardless of the type of movement, and sex, our results indicate that overall, addition of live *C. minuta* boosted physical activity.

One particularly striking effect of adding live *C. minuta* to the microbiome in both sexes was the increase in gut microbial biomass. The additional *C. minuta* cells were not themselves directly responsible for the higher microbial biomass. Rather, the metabolic activity of *C. minuta* in the microbiome was likely responsible. A greater microbial biomass has been associated with human health in the context of inflammatory bowel disease (40) and has been shown to be tightly connected to energy homeostasis in mice (40, 41) and in humans (42). Microbial fermentation, which supplies energy to the host, has been shown to be proportional to gut microbial biomass (19, 43). In humans, caloric intake has been shown to modulate the microbiome in a way that alters microbial biomass and diet energy extraction efficiency (44). Recently, Corbin showed in a well-controlled human study that a diet rich in fermentable fiber increased microbial biomass and increased energy loss in feces, but did not change host food intake or energy expenditure (45). In our study, the diet stayed the same, but alteration of the microbiome including higher microbial biomass was similarly associated with greater energy lost in feces. Microbial biomass is shed in feces, thus, the elevated microbial biomass itself may constitute a significant source of energy loss. By boosting the growth of other microbes in the gut, which then immobilize carbon in microbial biomass that is excreted with stool, a high level of *C. minuta* may be driving energy loss from the animal overall.

The association between *C. minuta* levels and biomass was unexpected and remains to be explained. *C. minuta* is an anaerobic bacterium that produces copious H_2_ and CO_2_ from the fermentation of sugars (46). We have previously shown that in the human gut, the family *Christensenellaceae* forms the hub in a co-occurrence network with other gut microbes (3). We know that *C. minuta* can act as a sole source of H_2_ and CO_2_ for methanogens in culture (7). Furthermore, we observed that *C. minuta* produced more H_2_ in culture than the gut bacterium *Bacteroides thetaiotaomicron* under similar conditions (7). We can assume that increasing *C. minuta* levels in the microbiome leads to higher levels of H_2_. A wide diversity of gut microbes metabolize H_2_ (47), such that H_2_ from *C. minuta* may boost their growth. Indeed, hydrogen administration to the gut microbiome in drinking water has been shown to alter gut microbial diversity (48, 49). Mice receiving hydrogen-water have also been shown to produce greater levels of SCFAs in the cecum (50), which is consistent with our observations of greater cecal SCFAs in Live-CM treated female mice. The H_2_ production capacity of *C. minuta* is thus one potential means by which it may standardize the diversity of the microbiome and shift metabolic output.

This work is very similar to previous reports with important differences in methodology and outcome. Previous work showed that addition of live *C. minuta* (3, 13) or a related species (12) to the microbiome decreased host adiposity gain in either female Swiss Webster (3) or male C57BL/6J mice (12, 13). We used both male and female Swiss Webster mice but did not observe effects on adiposity gains. We used the same donor stool as Goodrich et al. (3), although the stool was preserved for close to 10 years at −80°C between studies, which may be one factor that accounts for the difference in phenotype observed. Other differences may include differences in housing, specific mouse lineage, specific diet, and gavage frequency. Mazier et al. (13) reported reduced feed efficiency in their study, which we also observed here. Reduced feed efficiency, or the amount of body weight per food consumed, is consistent with our observations of both reduced energy harvest from the diet and higher levels of activity.

One important caveat is that housing conditions in our experimental design were confounded with sex for animal welfare reasons. Thus, the differences in the specific activity types that differed with treatment for male and female mice could be ascribed to their being caged singly or in groups, which affects their ambient temperature. Furthermore, individual housing potentially affects murine behavior and activity, as emerging literature suggests effects on the brain (51), sleep (52), and other factors (53). In addition, we measured host energy expenditure with a respirometry system, which estimates energy expenditure by measuring O_2_ consumption and CO_2_ production. While this method captures aerobic energy expenditure, nitrogenous and anaerobic processes are not reflected in the changes of O_2_ or CO_2_ (54), and as a result our estimation of energy expenditure with respirometry is incomplete. To acquire these energy expenditure-related measurements, we moved the mice for the last 3 days to a respirometry cage system. Despite using home cages during this period, we cannot exclude the possibility that our experimental design introduced an additional, uniform stressor to the mice before the endpoint of the study, possibly affecting our measurements. Our study consisted of approximately 45 mice per group for each sex, which is compararively large for fecal transfer experiments in mice; however we assessed that using linear mixed models with covariates such as weight (55), sex, and random effects (batch), this sample size is necessary to reveal small treatment effects (3). Finally, although our study comes a step closer to a mechanistic understanding of how *C. minuta* can alter host energy metabolism, more work is needed to understand the chain of events and molecular mechanisms involved.

### Prospectus

Our study shows that just one bacterial species, added to the microbiome, can result in altered voluntary behavior patterns in mice, but perhaps more importantly, has an outsized effect on the biomass of the gut microbiome. The present work adds to growing understanding that certain microbial taxa can act as keystones in the gut ecosystem. Keystone species need not be highly abundant in the community to exert a modifying influence on the diversity and function of the community with knock-on effects on the host. Whether the boost in biomass, or the associated changes to the metabolic output of the community, are responsible for the changes in energetics we observed in the host remains to be better understood.

## MATERIALS AND METHODS

### Animal experiments

All animal experimental procedures were reviewed and approved by the German Regierungspräsidium (animal experiment nr: EB 09/19 G). We purchased female and male 5–6-week-old GF Swiss Webster mice from Taconic Biosciences Inc. (Hudson, NY) or bred them in our facility.

#### Inoculation

We inoculated all mice with 200 µL of fecal slurry amended with living or heat-killed *C. minuta* by oral gavage. The fecal sample was previously described (3) and contained 0.003%–0.013% of *C. minuta* determined by two independent DNA extractions and phylogenetic profiling of shotgun sequences. To prepare the inoculum, we obtained *C. minuta* (DSM 22607) from the German Collection of Microorganisms and Cell Cultures (DSMZ; Braunschweig, Germany). We grew 2 × 80 mL of *C. minuta* in brain heart infusion media supplemented with yeast extract (5 g/L), reduced with L-cysteine-HCl (0.5 g/L), at 37°C under anaerobic conditions without shaking for 3 days. For the control group, the culture was heat-killed by autoclaving (20 min at 121°C) just before the preparation of the inoculum. In an anoxic glove box, we resuspended 0.3 g of stool in 4 mL of anaerobic PBS that contained 2 mM DTT by 5 min of vortexing. Both live and heat-killed *C. minuta* cultures were pelleted by centrifugation and added to half of the resuspended feces. We plated the remaining media of both live and heat-killed *C. minuta* and observed no growth in the latter group. The final inoculum contained approximately 1 × 10^10^ live or heat-killed *C. minuta* cells per mouse.

#### Housing

We performed experiments with 16 mice of the same sex, with 8 mice per treatment group. For each sex, we replicated the experiment six times, resulting in a combined number of 12 experiments with 192 mice (96 males, 96 females) in total. This number of mice is based on power calculations: based on the results of our previous experiments, we expected a small effect size with a reduction in adiposity gain of approximately 4%. Because of sickness or death, we excluded eight mice (one female Killed-CM sickness; one female Live-CM sickness; two males Live-CM death; two males Killed-CM death; two males Killed-CM sickness). All experiments were conducted in an in-house murine pathogen-free facility. For the first 25 days post-inoculation, we housed all mice at 22°C under a 12-h light/dark cycle in Digital Ventilated Cages (Tecniplast, Buguggiate, Italy), which recorded murine activity. We co-housed female mice from the same treatment group in groups of four, whereas male mice were single-housed (for their welfare). Autoclaved water and autoclaved polysaccharide-rich chow (Altromin, NIH31M) were provided *ad libitum*. During the weekly cage change, we monitored murine body weight. On day 25 post-inoculation, we transferred the mice into the behavioral phenotyping respirometry cage system (Promethion Sable Line, Nevada, USA), in which all mice were single-housed at 26°C under a 12-h light/dark cycle. Here, we monitored murine body weight, activity, behavior, food and water intake, oxygen consumption, and carbon dioxide production using a respirometry system. EE was calculated using the Weir equation (56). On day 28 post-colonization, the mice were euthanized, half by CO_₂_ and the other half by decapitation. Before euthanization, we fasted the mice for 5 h. Tissues were immediately collected, flash-frozen, and stored at −80°C. Blood was directly collected after decapitation. All blood samples were coagulated for 20 min at room temperature, centrifuged at 12,000*g* for 20 min at 4°C, after which the supernatant was collected and flash-frozen.

#### Body composition

Before inoculation and at day 25 post-inoculation, we measured fat mass and lean mass in the mice by quantitative magnetic resonance using an EchoMRI-100 (Echo Medical Systems, LLC., Texas, USA).

#### Bomb calorimetry

Gross energy content of fecal samples from mice was analyzed with the calorimeter bomb IKA C5003 (IKA-Werke GMBH & Co. KG, Staufen, Germany) at the Center of PhenoGenomics of the EPFL (Switzerland). Prior to the adiabatic measurement, the samples were dried under a PSM class II hood overnight. The calibration of the machine was performed with 0.5 g of benzoic acid.

### Metagenomics and qPCR

#### gDNA extraction

We isolated genomic DNA from frozen mouse cecal contents and aliquots of the gavage preparation (inoculum), using the PowerSoil htp DNA isolation kit (Qiagen, Valencia, CA, USA). We assessed the wet weight of cecal input samples used for DNA extraction before loading to the extraction plate.

#### Quantification by qPCR

We quantified genome equivalents from all bacteria and *C. minuta* in cecal genomic DNA by qPCR. We used the the KiCqStart SYBR Green qPCR ReadyMix and general 16S rRNA primers (515 forward: 5′-TCG TCG GCA GCG TCA GAT GTG TAT AAG AGA CAG GTG CCA GCM GCC GCG GTA A-3′, 806 reverse: 5′-GTC TCG TGG GCT CGG AGA TGT GTA TAA GAG ACA GGG ACT ACH VGG GTW TCT AAT-3′) or primers specific for *C. minuta* (27) (forward: 5′-TTC GGG AGG AAC TGT GGG TAT-3′, reverse: 5′-GGT TGC TCA CGC GTT ACT CA-3′). For the standard curve, we used genomic DNA from *Blautia hydrogenotrophica* or *C. minuta* extracted from pure cultures quantified with a Qubit 3.0 Fluorometer (high sensitivity assay kit). The total reaction volume per well was 20 µL with a primer concentration of 200 nM. The DNA volume per well was 3 µL, which we diluted priorly 1:100 for the quantification of *C. minuta* or all bacteria. We prepared the master-mixes and sample dilutions manually, but loaded the 384-well plates robotically (Tecan 780 Robot Fluent 780 Base Unit). The qPCR run was performed in the BioRad CFX384 Touch Real-Time PCR Detection System. The cycling conditions consisted of a 3-min incubation at 95°C, followed by a total of 40 cycles of 95°C incubations for 10 s, 10 s annealing at 55°C, and extension at 72°C for 30 s. Annealing temperature differed between the primers: general 16S rRNA = 55°C; C*. minuta* = 56.5°C. After the PCR melting, curve analysis was performed from 55°C to 95°C (5 s). Data were analyzed by Bio-Rad CFX96 Manager (v.3.1.1517.0823). We normalized the results by the wet weight of cecal content used for the DNA extraction.

#### Metagenomic sequencing

We prepared shotgun metagenome libraries with a modified Nextera protocol, as described elsewhere (57). Briefly, Nextera transposome, tagmentetd gDNA (5 ng) was amplified, and adapter sequences were added. Amplified libraries were purified with Angencourt AMPure XP beads (Beckman Coulter, Brea, CA, USA), followed by standard normalization and pooling of the samples. Fragments in sizes from 350 to 700 bp were selected by BluePippin (Sage Sciences). We ran 1 μL of the pooled libraries on Agilent 2100 Bioanalyzer with an HS DNA kit to check library size. We sequenced the barcoded pools on an Illumina HiSeq3000 or NextSeq2000 instrument with 2 × 150 bp paired-end sequencing. Library preparation and sequencing were performed at the Max Planck Institute for Biology Tübingen, Tübingen, Germany.

#### Sequence quality control

For the validation of the raw reads, we used fqtools v.2.0 (58). Next, we de-duplicated the reads with the clumpify module of bbtools v.37.78 (https://jgi.doe.gov/data-and-tools/bbtools/). Adapter trimming and read quality control were performed with skewer v.0.2.2 (59) and the bbduk module of bbtools. We filtered reads mapping to the human or mouse genome out by using the bbmap module of bbtools. Finally, fastqc v.0.11.7 (https://github.com/s-andrews/FastQC) and multiQC v.1.5a (60) generated QC reports for all reads.

#### Metagenomic profiling

Post-QC reads were taxonomically profiled with Kraken v.2.0 (61) and Bracken v.2.2 (62) against the custom databases generated using the Struo2 pipeline (63) based on GTDB release 202 (available at http://ftp.tue.mpg.de/ebio/projects/struo2/GTDB_release202/). We rarefried the microbial reads to 460,000 sequences, calculated relative frequencies from the rarefied reads, and multiplied them with the genome equivalents per gram cecal content to obtain an estimation of microbial absolute abundance.

### Metabolomics

#### Targeted serum metabolites

We measured 11 selected metabolites known to be modulated by the gut microbiome and affecting host behavior in the collected serum samples via LC-MS (Table S2). Serum metabolites were extracted by mixing pre-chilled methanol containing isotopic standards (^13^C_11_ L-tryptophan, kynurenic acid ring-D_5_) with serum at the ratio of 2:1 (v:v). The mixture with the final concentration of isotopic standards of 0.5 µg/mL was cooled for 30 min at −20°C before centrifuging at 11,000*g* at 4°C for 15 min. The supernatant was filtered through a regenerated carbon filter with a pore size of 0.2 µm. LC-MS analysis of metabolites of interest was performed using an HPLC system (Dionex UltiMate 3000, Thermo Fisher, USA) coupled with a high-resolution mass spectrometer with an electrospray ionization source (Impact II, Bruker, Germany). Reverse phase chromatography was executed using a C-18 column (*L* × inner diameter 250 cm × 4.6 mm, 5 µm particle size, Agilent P/N 990967-902) held at a constant temperature of 20°C. The mobile phase consisted of solvent A (0.1% formic acid in water) and solvent B (0.1% formic acid in acetonitrile). Gradient elution was applied with the following conditions: the chromatographic gradient started at 3% B for 3 min, increased linearly to 100% B at 20 min, held at 100% B for 3 min, decreased linearly to 3% B at 27 min, followed by an equilibrating time of 3 min. The flow rate was kept constantly at 0.5 mL/min. The injection volume was 40 µL. Samples were stored in the autosampler at 8°C for a maximum period of 72 h prior to injection. A dilution series of standards with a concentration range from 0.000128 to 10 µg/mL was measured to build the linear regression model. The weighting factor of 1, 1/*x*, or 1/*x*^2^ was selected depending on lowest sum percent relative error (64). The operating parameters of the mass spectrometer were as follows: the spray needle voltage at 3.5 kV, nitrogen was used as nebulizing gas (1.5 bar) and drying gas (5 L/min), and the drying temperature was at 200°C. The data were acquired in Full-MS mode with a scanning range of 50–1,000 *m*/*z* and a scanning rate of 2 Hz in the positive ion mode. Each measurement included a 30-s segment for automated internal calibration using sodium formate 5 mM. Peak integration of Full-MS data was performed using Skyline (v.21.1). The internal isotopic standards were used for quality control and normalization purposes.

#### Untargeted pathway analysis

We analyzed our data with XCMS (v.2.7.2)(31), MaAsLin2 (29), and MetaboAnalyst5.0 (www.metaboanalyst.ca) (32). Briefly, LC-MS raw data files were converted into the mzML format using ProteoWizard MS converter (v.3.0) applying peak picking on MS1 level (65) and then processed by XCMS software (v.2.7.2) to perform peak extraction, baseline calibration, peak alignment, peak identification, retention time (RT) correction, and integration of peak area. The parameters setting was as follows: centWave method setting for feature detection (ppm = 20, peak width = 12.8–80 s, signal/noise threshold = 10, mzdiff = −0.001, prefilter intensity = 100, and prefilter peaks = 3); Obiwarp method setting for RT correction (ProfStep = 0.5), parameters for chromatogram alignment (mzwid = 0.01, minfrac = 1, bw = 1, and minsamp = 1). The output data matrix including RTs, *m*/*z* values, and peak intensity was further analyzed with MaAsLin2, correcting for batch effects. The calculated *t* values and multiple hypothesis corrected *P* values (Benjamini-Hochberg) for each *m*/*z* and RT pair were exported and analyzed with the functional analysis in MetaboAnalyst5.0. The parameters setting was as follows: ion mode: positive; mass tolerance: 5 ppm; algorithms: GSEA and mummichog v.2; *P* value cutoff: 0.05; adducts: M^+^, [M + H]^+^, [M + 2H]^2+^, [M + Na]^+^, [M + K]^+^, [M − H_2_O + H]^+^, [M + NH_4_]^+^, [2M + H]^+^; pathway library: *Mus musculus* (KEGG). Pathways with a combined *P* value smaller than 0.05 were considered significant.

#### Cecal SCFAs

The samples and SCFA standard mix tubes were treated as reported by Furuhashi et al. with few modifications (66). Briefly, 3-methyl pentanoate was added as an internal standard. Then, 125 µL of 20 mM NaOH, 100 µL of pyridine, and 80 µL of isobutanol were added, and the final volume was adjusted to 650 µL with ultrahigh-quality water. Next, we derivatized the solution with 50 µL of isobutyl-chloroformate and kept the lid open for 1 min to release generated gasses. The sample was vortexed for 30 s, spun down, and 150 µL of hexane was added. We vortexed the sample for 10 s and centrifuged it at 21,000 × *g* for 3 min. Thereafter, we transferred the upper hexane phase into an autosampler vial. One microliter of the sample was injected into gas chromatography-mass spectrometry (GC-MS) in split mode (1:50) with helium as a carrier gas at a flow rate of 1 mL/min. Measurements were carried out on a single quadrupole mass spectrometer (5977B-MSD) equipped with 7890B GC and 7693 autosampler, all from Agilent Technologies, Santa Clara, CA, USA. We set the temperature of the GC-MS ion source and the transfer line for the samples to 280°C. A VF-5ms column (60 m, 0.25 mm, 0.25 µm; CP8961, Agilent, USA) was used. The oven temperature gradient for the samples was as follows: after 5 min at 40°C, the oven was programmed to rise to 300°C at a rate of 10°C/min. We set the temperature of the GC-MS ion source to 250°C and the transfer line to 350°C. The scan range was between *m*/*z* 30 and 600. A 70 eV EI mode (Extractor ion source; Agilent Technologies, Santa Clara, CA, USA) was used, and SCFAs were quantified by peak areas’ estimations in the extracted ion chromatogram (MassHunter; Agilent Technologies, Santa Clara, CA, USA).

### Statistical analyses

We applied linear mixed models to analyze the statistical difference using lmerTest (67) and normalized values with the bestNormalize package (68). Here, we corrected for the batch of the experimental replicates, sex, technical variables and in case of EE, food intake and fecal energy loss additionally for murine weight (69). In the case of analyzing two continuous variables, we extracted marginal and conditional *R*^2^ values. Furthermore, we used simple Spearman’s correlation analyses for cases in which the covariates and random effects would have been completely shared for the predictor and the explanatory variable (Fig. 3B; Fig. S3A and S4B). To avoid normality problems, we applied Wilcoxon rank sum tests for the analyses of α-diversity metrics and permuted Wilcoxon rank sum tests for intra-group UniFrac distances. Results with a *P* value <0.5 were considered significant.

In the case of the targeted metabolites in murine sera, SCFAs in murine cecal contents, and microbial species, we used MaAsLin2 (29) to perform differential abundance analyses followed by a multiple-hypothesis correction (Benjamini-Hochberg). In the case of the analyses of microbial species, we considered taxa with a median relative abundance lower than 0.00001 as absent and set the prevalence threshold for taxa to 0.5. Metabolites or species with a *q* < 0.1 were considered significant. Statistical tests are indicated in figure legends.

## Data Availability

Sequencing reads are deposited in the European Nucleotide Archive (ENA) under the following project accession: PRJEB66907. The R code and metadata are deposited in https://github.com/leylabmpi/Schoen_Christensenella_2023. Metabolome data are accessible as follows: GCMS data have the MassIVE ID: MSV000093076 and LCMS data have the MassIVE ID: MSV000093077.

## SUPPLEMENTAL MATERIAL

Fig. S1 mbio.02836-23-s0001.eps Global map of studies associating the gut bacterial family *Christensenellaceae* with healthy body mass index and/or metabolic health in human gut microbiome studies.

Fig. S2 mbio.02836-23-s0002.eps Murine body weight gain and fecal mass.

Fig. S3 mbio.02836-23-s0003.eps Mouse voluntary activity and average resting metabolic rate.

Fig. S4 mbio.02836-23-s0004.eps *C. minuta* amendment resulted in lower complexity in males.

Fig. S5 mbio.02836-23-s0005.eps Candidate metabolites assessed in cecal contents and serum samples.

Fig. S6 mbio.02836-23-s0006.eps Groups defined by *C. minuta* abundance revealed a trend for lower fecal energy content and a trend for higher overall activity in both sexes.

Fig. S7 mbio.02836-23-s0007.eps Higher microbial biomass and lower α-diversity in mice with a high *C. minuta* abundance.

Supplemental material mbio.02836-23-s0008.docx Tables S1 to S4 and supplemental figure.

ASM does not own the copyrights to Supplemental Material that may be linked to, or accessed through, an article. The authors have granted ASM a non-exclusive, world-wide license to publish the Supplemental Material files. Please contact the corresponding author directly for reuse.
