# Supplementary material for: The keystone gut species Christensenella minuta boosts gut microbial biomass and voluntary physical activity in mice
Source: mBio. 2023 Dec 22;15(2):e02836-23. doi: 10.1128/mbio.02836-23 (PMC10865807; doi:10.1128/mbio.02836-23)
Supplement: Supplemental material — Tables S1 to S4 and supplemental figure. [file mbio.02836-23-s0008.docx]

**Supplement for Schoen et al:**

The keystone gut species *Christensenella minuta* boosts gut microbial biomass and voluntary physical activity in mice

**Table S1: Mean and se per α-diversity metric for each treatment group by sex**

| **α-diversity metric** | **Females** | | | **Males** | | |
| --- | --- | --- | --- | --- | --- | --- |
|  | ***Live-CM*** | ***Killed-CM*** | ***p*** | ***Live-CM*** | ***Killed-CM*** | ***p*** |
| # microbial species | 761 ± 48 | 796 ± 54 | 0.75 | 622 ± 25 | 785 ± 54 | 0.022 * |
| Faith’s PD | 145 ± 8 | 150 ± 9 | 0.61 | 126 ± 5 | 147 ± 8 | 0.047 * |
| Shannon Entropy | 4.78 ± 0.05 | 4.86 ± 0.05 | 0.4 | 4.36 ± 0.04 | 4.63 ± 0.07 | 0.003 ** |
| Pilou evenness | 0.51 ± 0.005 | 0.51 ± 0.004 | 0.25 | 0.47 ± 0.006 | 0.49 ± 0.006 | 0.14 |

**Table S2: Literature linking target metabolites to the gut-microbiome-brain axis.**

| **Pathway** | **Compounds** | **Literature** |
| --- | --- | --- |
| Hypothalamic–  pituitary–thyroid axis | T4 (thyroxine) | Probiotic administration increases locomotor activity and improves thyroid function [(Varian et al. 2014)](https://paperpile.com/c/6uowJJ/cRinO)  Probiotic administration increase levothyroxine availability and stabilizes thyroid function [(Spaggiari et al. 2017)](https://paperpile.com/c/6uowJJ/NHemC) |
| Hypothalamic–  pituitary–adrenal axis | Corticosterone | Probiotic administration alleviated depressive-like behaviors and decreased corticosterone level in mice subjected to restraint stress [(Liu et al. 2020)](https://paperpile.com/c/6uowJJ/hA0Y9)  Probiotic administration alleviated behavioral changes of germfree mice and modulated glucocorticoid pathway genes in the brain and serum cortisol concentrations [(Y. Luo et al. 2018)](https://paperpile.com/c/6uowJJ/HZcTt) |
| Histamine - Neurotransmitter | Histamine | Histamine is critical for learning, memory, cognition, and motivation [(Theoharides 2015)](https://paperpile.com/c/6uowJJ/T67h4)  Various gut microbes can produce histamine [(Chen et al. 2019)](https://paperpile.com/c/6uowJJ/6dRYC) |
| GABA - Neurotransmitter | γ-aminobutyric acid (GABA),  Glutamine,  Glutamate | Various probiotics can synthesize GABA, glutamine and glutamate in vitro and change GABA levels in murine feces [(Luck et al. 2021)](https://paperpile.com/c/6uowJJ/mk4xO)  Fecal transplant experiments from schizophrenic human donors resulted in lower GABA levels in the brain and schizophrenia-related behaviors in recipient mice compared to controls [(Zheng et al. 2019)](https://paperpile.com/c/6uowJJ/XbOe0)  Probiotic administration affects GABA receptor expression in the brain, reduces stress-induced corticosterone levels and anxiety- and depression-related behavior in mice [(Bravo et al. 2011)](https://paperpile.com/c/6uowJJ/IFShy)  Colonization of germfree mice normalized anxiety-like behavior and brain BDNF levels, associated with expression of the GluN2A subunit of the Glutamate Receptor [(Sudo et al. 2004)](https://paperpile.com/c/6uowJJ/yANvw) |
| Tryptophan metabolism | Kynurenine,  Kynurenic acid, Tryptophan, Indolelactic acid, Serotonin | High dosage of tryptophan decreased murine activity [(Modigh 1973)](https://paperpile.com/c/6uowJJ/iTqXY)  Serum serotonin and tryptophan metabolite concentrations depend on host colonization status [(O’Mahony et al. 2015)](https://paperpile.com/c/6uowJJ/KMQ8k)  Probiotic administration improved cognitive and anxiety-like behavior and affected central serotonin levels as well as metabolites of the kynurenine pathway [(J. Luo et al. 2014)](https://paperpile.com/c/6uowJJ/kY5Na) |

**Table S3: Predicted serum metabolites for pathways significantly associated with EE in female mice using MetaboAnalyst5.0.**

| **Pathway**  (significant hits/  total hits/  total # of metabolites in pathway) | **predicted metabolites KEGG** | **Name** | **detected match** | **significant hit** |
| --- | --- | --- | --- | --- |
| **Steroid hormone biosynthesis**  (11/30/77) | C01953 | Pregnenolone | x |  |
|  | C05487 | 17alpha,21-Dihydroxypregnenolone | x |  |
|  | C05489 | 11beta,17alpha,21-Trihydroxypregnenolone |  | x |
|  | C02140 | Corticosterone |  | x |
|  | C00410 | Progesterone | x |  |
|  | C05476 | Tetrahydrocorticosterone | x |  |
|  | C05498 | 11beta-Hydroxyprogesterone |  | x |
|  | C03681 | 5alpha-Dihydroprogesterone | x |  |
|  | C00735 | Cortisol |  | x |
|  | C05284 | 11beta-Hydroxyandrost-4-ene-3,17-dione | x |  |
|  | C05488 | 11-Deoxycortisol |  | x |
|  | C05497 | 21-Deoxycortisol |  | x |
|  | C00280 | Androstenedione | x |  |
|  | C00951 | Estradiol | x |  |
|  | C05299 | 2-Methoxyestrone | x |  |
|  | C05302 | 2-Methoxy-17beta-estradiol | x |  |
|  | C01124 | 18-Hydroxycorticosterone |  | x |
|  | C05290 | 19-Hydroxyandrostenedione | x |  |
|  | C05297 | 19-Oxoandrostenedione | x |  |
|  | C05295 | 19-Oxotestosterone | x |  |
|  | C18044 | Pregnenolone sulfate | x |  |
|  | C05140 | 4-Androsten-16alpha-ol-3,17-dione | x |  |
|  | C05473 | 11beta,21-Dihydroxy-3,20-oxo-5beta-pregnan-18-al |  | x |
|  | C04042 | 20alpha-Hydroxyprogesterone | x |  |
|  | C05490 | 11-Dehydrocorticosterone |  | x |
|  | C05478 | 5beta-Pregnane-3alpha,21-diol-11,20-dione | x |  |
|  | C05479 | 5beta-Pregnane-3,20-dione | x |  |
|  | C05471 | Dihydrocortisol |  | x |
|  | C05469 | 4,5beta-Dihydrocortisone |  | x |
|  | C05285 | Adrenosterone | x |  |
| **Primary bile acid biosynthesis**  (2/4/46) | C00245 | Taurine | x |  |
|  | C00695 | Cholic acid |  | x |
|  | C05466 | Glycochenodeoxycholate |  | x |
|  | C05122 | Taurocholate | x |  |
| **Fatty acid biosynthesis**  (1/2/10) | C01571 | Decanoic acid | x |  |
|  | C06423 | Octanoic acid |  | x |
| **Porphyrin and chlorophyll metabolism**  (2/7/27) | C15672 | Heme O | x |  |
|  | C00500 | Biliverdin |  | x |
|  | C01079 | Protoporphyrinogen IX | x |  |
|  | C00931 | Porphobilinogen | x |  |
|  | C00430 | 5-Aminolevulinate | x |  |
|  | C00025 | L-Glutamate | x |  |
|  | C00486 | Bilirubin |  | x |

**Table S4: Mean and se per α-diversity metric for the *High-CM* and *Low-CM* groups**

| **α-diversity metric** | ***High-CM*** | ***Low-CM*** | ***p*** |
| --- | --- | --- | --- |
| # observed species | 835 ± 38 | 646 ± 24 | 1.6 e^-4^ *** |
| Faith’s PD | 126 ± 4 | 158 ± 6 | 1.3 e^-4^ *** |
| Shannon Entropy | 4.6 ± 0.04 | 4.7 ± 0.05 | 0.48 |
| Pilou evenness | 0.5 ± 0.004 | 0.49 ± 0.004 | 0.13 |

**Figure legends**

**Figure S1 - Global map of studies associating the gut bacterial family *Christensenellaceae* with healthy body mass index and/or metabolic health in human gut microbiome studies.** Sampling locations of 42 studies associating the abundance of *Christensenellaceae* in the gut with a healthy BMI (pink) or metabolic health (green) in humans to the world map [1–42]. Circle size indicates study cohort size.

**Figure S2 - Murine body weight gain and fecal mass.** (A) Mouse weight gain over the course of the experiment. (B) Residuals of daily fecal mass excreted during day 25 and 28 post inoculation adjusted for weight, sex and batch. Adj. = adjusted.

**Figure S3 - Mouse voluntary activity and average resting metabolic rate.** (A) Average running speed per treatment group. (B) Residual average RMR adjusted for weight, sex and batch measured by indirect calorimetry. Adj. = adjusted.

**Figure S4 - *C. minuta* amendment resulted in lower complexity in males.** (A-E) Analyses of phylogenetically-profiled metagenomic cecal sequences normalized by microbial biomass quantified via qPCR. (A) Taxa bar plots for each mouse. (B-C) α-diversity metrics between treatment groups. Asterisks indicate statistical significance of Wilcoxen rank sum test. * : p < 0.1; *** : p < 0.001. GE = genome equivalents.

**Figure S5 - Candidate metabolites assessed in cecal contents and serum samples.** (A-C) SCFA concentrations in murine cecal contents on day 28 post-inoculation measured via GC-MS. (A) Individual SCFA per treatment. (B) Sum of all SCFA and (C) butyrate concentrations in correlation to murine overall activity. (D) Targeted metabolites in murine sera measured via LC-MS. (B-C) Marginal *R^2^*- and *p*-values of linear mixed models are stated in the panels. Adj. = adjusted; GABA = γ-Aminobutyric acid; Gln = Glutamine; Glu = Glutamic acid; ILA = Indolelactic acid; K = *Killed-CM*; KYN = Kynurenine; KYNA = Kynurenic acid; L = *Live-CM*; SCFA = short chain fatty acid; T4 = Thyroxine.

**Figure S6 - Groups defined by *C. minuta* abundance revealed a trend for lower fecal energy content and a trend for higher overall activity in both sexes.** (A) Grouping of mice according to *C. minuta* quantification in murine cecal contents on day 28 post-inoculation by qPCR. Threshold set at median abundance of *C. minuta* (median = 11072203 GE; *C. minuta* abundance < median: *High-CM* group; *C. minuta* abundance >= median: *Low-CM* group). Murine (B) daily food intake from day 25 to day 28 post-inoculation and (C) feed efficiency, calculated with daily weight gain from from day 0 to day 28 post-inoculation and daily food intake. (D) Fecal energy loss between day 25 and 28 post-inoculation. (E-F) Activity measurements, (E) overall activity and (F) locomotion, in the metabolic cage system per group. (B-F) Residuals adjusted for sex, batch and (B, D) murine weight. Asterisks indicate statistical significance of the linear mixed model correcting for sex, batch and (B, D) mouse weight. * : p < 0.1. Adj. = adjusted.

**Figure S7 - Higher microbial biomass and lower α-diversity in mice with a high *C. minuta* abundance.** (A-B) Quantification of microbial biomass via qPCR with universal 16S rRNA primers (A) by group and (B) in association to fecal energy content. (C) Association to fecal energy content to the abundance of *C. minuta.* (D-G) α-diversity of phylogenetically-profiled metagenomic cecal sequences by group. (H-I) Intra-group (H) weighted and (I) unweighted UniFrac distances. Box plots depict 25 and 75 % quantiles, with a horizontal line at the median, whiskers marking the 1.5 interquartile range. Asterisks indicate statistical significance of the (A) linear mixed model correcting for sex and batch or (D-I) the Wilcoxen rank sum test. (B-C) Marginal *R^2^*- and *p*-values of linear mixed models are stated in the panels. * : p < 0.1; ** : p < 0.01; *** : p < 0.001; Adj: =adjusted; GE = genome equivalents; Norm. = normalized.

**Supplementary References**

[Bravo, Javier A., Paul Forsythe, Marianne V. Chew, Emily Escaravage, Hélène M. Savignac, Timothy G. Dinan, John Bienenstock, and John F. Cryan. 2011. “Ingestion of *Lactobacillus* Strain Regulates Emotional Behavior and Central GABA Receptor Expression in a Mouse via the Vagus Nerve.” *Proceedings of the National Academy of Sciences of the United States of America* 108 (38): 16050–55.](http://paperpile.com/b/6uowJJ/IFShy)

[Chen, Haiwei, Phu-Khat Nwe, Yi Yang, Connor E. Rosen, Agata A. Bielecka, Manik Kuchroo, Gary W. Cline, et al. 2019. “A Forward Chemical Genetic Screen Reveals Gut Microbiota Metabolites That Modulate Host Physiology.” *Cell* 177 (5): 1217–31.e18.](http://paperpile.com/b/6uowJJ/6dRYC)

[Liu, Quan Feng, Hong-Man Kim, Sanghyun Lim, Myung-Jun Chung, Chi-Yeon Lim, Byung-Soo Koo, and Seok-Seong Kang. 2020. “Effect of Probiotic Administration on Gut Microbiota and Depressive Behaviors in Mice.” *Daru: Journal of Faculty of Pharmacy, Tehran University of Medical Sciences* 28 (1): 181–89.](http://paperpile.com/b/6uowJJ/hA0Y9)

[Luck, Berkley, Thomas D. Horvath, Kristen A. Engevik, Wenly Ruan, Sigmund J. Haidacher, Kathleen M. Hoch, Numan Oezguen, et al. 2021. “Neurotransmitter Profiles Are Altered in the Gut and Brain of Mice Mono-Associated with Bifidobacterium Dentium.” *Biomolecules* 11 (8). https://doi.org/](http://paperpile.com/b/6uowJJ/mk4xO)[10.3390/biom11081091](http://dx.doi.org/10.3390/biom11081091)[.](http://paperpile.com/b/6uowJJ/mk4xO)

[Luo, Jia, Tao Wang, Shan Liang, Xu Hu, Wei Li, and Feng Jin. 2014. “Ingestion of Lactobacillus Strain Reduces Anxiety and Improves Cognitive Function in the Hyperammonemia Rat.” *Science China. Life Sciences* 57 (3): 327–35.](http://paperpile.com/b/6uowJJ/kY5Na)

[Luo, Yuanyuan, Benhua Zeng, Li Zeng, Xiangyu Du, Bo Li, Ran Huo, Lanxiang Liu, et al. 2018. “Gut Microbiota Regulates Mouse Behaviors through Glucocorticoid Receptor Pathway Genes in the Hippocampus.” *Translational Psychiatry* 8 (1): 187.](http://paperpile.com/b/6uowJJ/HZcTt)

[Modigh, K. 1973. “Effects of L-Tryptophan on Motor Activity in Mice.” *Psychopharmacologia* 30 (2): 123–34.](http://paperpile.com/b/6uowJJ/iTqXY)

[O’Mahony, S. M., G. Clarke, Y. E. Borre, T. G. Dinan, and J. F. Cryan. 2015. “Serotonin, Tryptophan Metabolism and the Brain-Gut-Microbiome Axis.” *Behavioural Brain Research* 277 (January): 32–48.](http://paperpile.com/b/6uowJJ/KMQ8k)

[Spaggiari, Giorgia, Giulia Brigante, Sara De Vincentis, Umberto Cattini, Laura Roli, Maria Cristina De Santis, Enrica Baraldi, et al. 2017. “Probiotics Ingestion Does Not Directly Affect Thyroid Hormonal Parameters in Hypothyroid Patients on Levothyroxine Treatment.” *Frontiers in Endocrinology* 8 (November): 316.](http://paperpile.com/b/6uowJJ/NHemC)

[Sudo, Nobuyuki, Yoichi Chida, Yuji Aiba, Junko Sonoda, Naomi Oyama, Xiao-Nian Yu, Chiharu Kubo, and Yasuhiro Koga. 2004. “Postnatal Microbial Colonization Programs the Hypothalamic-Pituitary-Adrenal System for Stress Response in Mice.” *The Journal of Physiology* 558 (Pt 1): 263–75.](http://paperpile.com/b/6uowJJ/yANvw)

[Theoharides, Theoharis C. 2015. “On the Gut Microbiome-Brain Axis and Altruism.” *Clinical Therapeutics* 37 (5): 937–40.](http://paperpile.com/b/6uowJJ/T67h4)

[Varian, B. J., T. Poutahidis, T. Levkovich, Y. M. Ibrahim, J. R. Lakritz, A. Chatzigiagkos, A. Scherer-Hoock, E. J. Alm, S. E. Erdman, and Others. 2014. “Beneficial Bacteria Stimulate Youthful Thyroid Gland Activity.” *Journal of Obesity & Weight Loss Therapy* 4 (2).](http://paperpile.com/b/6uowJJ/cRinO) <https://www.cabdirect.org/cabdirect/abstract/20153424026>[.](http://paperpile.com/b/6uowJJ/cRinO)

[Zheng, Peng, Benhua Zeng, Meiling Liu, Jianjun Chen, Junxi Pan, Yu Han, Yiyun Liu, et al. 2019. “The Gut Microbiome from Patients with Schizophrenia Modulates the Glutamate-Glutamine-GABA Cycle and Schizophrenia-Relevant Behaviors in Mice.” *Science Advances* 5 (2): eaau8317.](http://paperpile.com/b/6uowJJ/XbOe0)
